# Blood-derived non-extracellular vesicle proteins as potential biomarkers for the diagnosis of early ER+ breast cancer and detection of lymph node involvement

**DOI:** 10.12688/f1000research.14129.3

**Published:** 2018-05-10

**Authors:** Rod Tucker, Ana Pedro

**Affiliations:** 1 Roma Laboratories Ltd, Hull, East Yorkshire, HU7 3GE, UK

**Keywords:** ER+ breast cancer, extracellular vesicles, plasma, biomarkers, diagnostic, lymph node involvement, metastases

## Abstract

Extracellular vesicles (EV’s) are membrane surrounded structures released by different cell types and are emerging as potential therapeutic and diagnostic targets in cancer. In the present study, plasma samples derived from 7 patients with metastatic and non-metastatic ER+ (estrogen receptor positive) breast cancer (BC) were collected and their respective (EVs) isolated and the protein content analyzed by mass spectrometry and FunRich analysis. Two putative plasma biomarkers (absent in healthy controls samples) were identified which could be used to detect early ER+ breast cancer and for those with lymph node (LN) involvement However, given the current limitations of the EV isolation method used, it is possible that these biomarkers did not originate from EVs and may represent blood-derived extracellular proteins.  Further work in a larger patient cohort is warranted to confirm these findings and examine the diagnostic potential of these biomarkers.

Extracellular vesicles (EVs) are membrane surrounded structures released by different cell types that are involved in cellular communication and are emerging as potential therapeutic and diagnostic targets in cancer ^1^ as in the case of early pancreatic cancer ^2^.

EVs can be classified in several subtypes based on their size, shape, and supposed origin. Exosomes are defined as ∼30–100 nm vesicles which originate from multivesicular bodies (MVB) and contain late endosomal markers ^3, 4^, although biochemically indistinguishable vesicles can bud directly from the plasma membrane ^3, 5^. Microvesicles or shedding vesicles are generally larger (>200 nm), are more variable in shape and density, and likely originate from the plasma membrane ^4, 6, 7^. EVs may contain proteins, lipids, and RNAs, however how these components are sorted into EVs remains unclear.

Tumor-derived EVs are also critical components for preparing the tumor microenvironment because they enable tumor cells to escape from the immunological surveillance ^8^ and help in the setting of a pre-metastatic niche for the engraftment of detached cancer cells ^9^. Both exosomes and MVs have been extensively studied and attributed various important physiological roles in cancer ^10, 11^. For instance, EVs have been found to play an important role in every phase of cancer development from cancer initiation, invasion and metastasis ^12^. For these reasons, EVs are potential therapeutic and diagnostic targets in cancer and EV-derived biomarkers maybe useful for predicting future metastatic development and identify metastasis sites ^13^.

ER+ (estrogen receptor positive) breast cancer (BC) represents 60–80% of all BC cases ^14, 15^. Here we describe our preliminary findings exploring the role of tumour derived EVs biomarkers that could ultimately be used as part of a test kit for the detection of early ER+ BC and lymph node involvement.

## Methods

### Samples

Plasma samples from 4 control patients (2 adult women and 2 men) which were confirmed as not having any form of BC, ER+ BC metastases, BC1 and BC2 explants EVs, SKBC and parental BC (Lyden lab, WCM, USA). Samples CF37, CF5, CF1, CF25, CF33, CF27 and CF110 and C7 (female control plasma sample) were collected at Champalimaud Clinical Centre, Portugal, as part of a study on the role of tumor-derived microvesicles and bone marrow progenitor cells as diagnostic and prognostic biomarkers in advanced BC and inflammatory BC Patients (RECI/BIM-ONC/0201/2012, FCT, Portugal). ER+ BC patient samples were selected based on their stage of disease progression – confirmed by CT-scan and surgery. EVs derived from conditioned media of cells lines SKBr3, MCF7, MDA468, MDA231 and MCF10A were also used in this study (details about these samples can be found in Table 1).

**Table 1.** Clinical data for different patient samples and cell lines.

Sample ID	Menopausal status	ER/PR/Her2 status (%)	Metastases pattern	Sample type
CF5	pre	100/95/-	LN+	Plasma
CF37	pos	100/-/-	LN-	Plasma
CF110	pos	100/100/-	Locally advanced	Plasma
CF1	pre	100/100/-	LN, liver	Plasma
CF25	pos	75/25/-	LN, liver, cartilage, skin	Plasma
CF33	pos	100/?/-	LN, liver, bone, skin, lung, brain	Plasma
CF27	pos	100/1/-	LN, lung, bone	Plasma
SKBC	?	?	Multiple metastasis	Plasma
BC1	?	ER+	Bone	Bone metastasis explant conditioned media
BC2	?	ER+	Bone	Bone metastasis explant conditioned media
Parental breast cancer	?	?	Primary tumor	Primary breast cancer conditioned media
SKBr3 (metastatic in mice) ^20^	? (43y)	HER2+	Metastasis	Pleural effussion (ATCC) Conditioned media from cell line culture
MDA468 (metastatic in mice) ^21^	? (51y)	TN (triple-negative)	Metastasis	Pleural effussion (ATCC) Conditioned media from cell line culture
MDA231 (highly metastatic in mice)	? (51y)	TN	Metastasis	Pleural effussion (ATCC) Conditioned media from cell line culture
MCF7 (poorly metastatic in mice)	pos	ER+	Metastasis	Pleural effussion (ATCC) Conditioned media from cell line culture
MCF10A	pre	Benign -fibrocystic disease	------	Mammary gland; breast (ATCC) Conditioned media from cell line culture

### Ethics approval and informed consent

This study was approved by an Ethics Review Board at Champalimaud Foundation, Portugal. All study patients provided their written, informed consent.

### EV purification and analysis

EV purification and analysis were performed at the Lyden lab (WCM) accordingly to Andre *et al*., 2016 ^16^. Briefly, plasma was pelleted at 500 × *g* for 10 min, then the supernatant was centrifuged at 20,000 × *g* for 20 min. Exosomes were then harvested by centrifugation at 100,000 × *g* for 70 min. The exosome pellet is resuspended in PBS and collected by ultracentrifugation at 100,000 × *g* for 70 min. The exosome pellet is resuspended in PBS and then stored at −80°C. The LM10 nanoparticle characterization system (NanoSight) equipped with a blue laser (405 nm)

### Proteomics and proteomic analysis

Proteomic analysis was performed at the Rockefeller University, Proteomics Center as described in Hamidi *et al.*, 2017 ^17^. Proteomic analysis was performed with the help of FunRich Program version 3. Only proteins with Mascot scores of approximately 90 or >90 were considered ^18^.

## Results and discussion

Clinical data on the EVs isolated from BC patient’s plasma samples and cell lines can be found in Table 1. The method used for EV isolation also precipitates lipoproteins and immunocomplexes (IC) which are known possible contaminants ^19^. However, samples submitted for mass spectrometry analysis showed none of the recognised contaminants of high speed centrifugation. In the two patients with early BC ( Table 2a), we detected HCG1745306 isoform CRA-a, a protein from the family of alpha type haemoglobins and for the patient with lymph node involvement, we detected histone H1.2 ( Table 2 a–b). HCG1745306 isoform CRA-a was only present in the two patients with early BC with Mascot scores of 3208.8 and 3966.5, respectively and absent in all controls and other patient samples.

**Table 2.** a–b, Plasma EV-derived candidate biomarkers for early ER+ breast cancer and LN involvement. Also, represented the Mascot scores for each protein in each sample.

a											
	Female1	Female2	Male1	Male2	CF37 (LN-)	CF5 (LN+)	CF110	CF1	CF25	CF27	CF33
G3V1N2 (HCG1745306, isoform CRA_a) **Early breast cancer**	0	0	0	0	3208.75461	3966.542	0	0	0	0	0
P16403 (Histone H1.2) **Early** **breast cancer, LN involvement**	0	0	0	0	0	325.1718	0	0	0	0	0

b										
		**SKBC**	**BC1**	**BC2**	**Parental BC**	**SKBr3**	**MDA468**	**MDA231**	**MCF7**	**MCF10A**
P16403 Histone H1.2		0	638.4	117.38	102.9	154.45	427.1	90.35	NS	0

Histone H1.2 was also detected in samples from the two patients with bone metastases, a parental primary BC sample and metastatic SKBr-3, MDA468, MDA231 cell lines. However, histone H1.2 was absent from the plasma sample of a patient with multiple metastases, from the non-metastatic MCF7 cell line (a non significant mascot score) and from MCF10A cells EVs ( Table 2b). These observation suggests that histone H1.2 might represent a potential marker for LN involvement and metastatic potential. Recent studies suggest histone H1.2 phosphorylation may be useful as a clinical biomarker of breast and other cancers because of its ability to recognize proliferative cell populations. Both MCF7 (expressing an allelic variant A142T) and MDA231, have a greater number of histone H1.2 phosphorylations when compared to MCF10A cell line ^22^. Curiously, phosphorylation of histone H1.2 at S173 increases during the M phase relative to the S phase, suggesting that this event is cell cycle-dependent and may serve as a marker for proliferation of cancer cells during BC invasion ^23, 24^. Also, histone H1.2 is a novel component of the nucleolar organizer regions during mitosis ^25^ and H1.2 depletion was observed in a human BC cell line caused cell cycle G1-phase arrest ^26^. Indeed, a higher mitotic index (≥ 7) in primary tumors is significantly associated with LN involvement ^27^ and higher mitotic indices accurately predict axillary LN involvement at operation ^28^.

**Figure 1.**
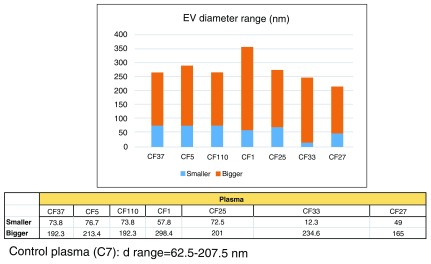
Nanosight (NTA) analysis for samples C7, CF37, CF5, CF110, CF1, CF25, CF27 and CF33.

Although we have identified two potential biomarkers possibly derived from EVs, our study does suffer from a number of recognised limitations. Firstly, ultracentrifugation is insufficient to purify EVs from other contaminants ^29^. For example, co-isolation of high-density lipoprotein and other particles with EVs isolated from blood by density gradient centrifugation has been reported ^29, 30^ suggesting that the biomarkers we identified might not be associated with EVs but with a constituent of another particle type such as a lipoprotein. Secondly, as mentioned above, exosomes are defined as ∼30–100 nm vesicles that originate from MVB. In contrast, microvesicles or shedding vesicles are generally larger (>200 nm), more variable in shape and density and arise from the plasma membrane. The size of the particles we isolated ranged from 76.7-213.4 and 73.8-192.3 nm, for samples CF5 and CF37, respectively and for all the samples between 12.3-298.4nm ( Figure 1 and original NTA files) possibly correspond to low-density lipoproteins which have the same size as EVs ^31^. Moreover, it is unlikely that EVs would contain a histone (which are normally confined to DNA in the nucleus). However, Thakur *et al.*, claim to have identified genomic DNA in EVs by electron microscopy (EM) though the EM image is not of sufficient magnification to allow for an accurate morphologic analysis and may simply represent cellular debris or apoptotic bodies or even unspecific staining ^32, 33^. Additionally, it is also unlikely HCG1745306 isoform CRA-a, would be present in EVs and it may simply be a precipitant similar to the α-globin seen in β-thalassemia ^34^. Therefore our current data does not support the idea that these biomarkers derived from EVs and could in fact be blood-derived extracellular proteins.

Nevertheless, a strength of our study is that samples were drawn from those with confirmed non-metastatic and metastatic disease at different sites and so are likely to be representative patients.

The mass spectrometry analysis results from all patient samples Click here for additional data file. Copyright: © 2018 Tucker R and Pedro A 2018 Data associated with the article are available under the terms of the Creative Commons Zero "No rights reserved" data waiver (CC0 1.0 Public domain dedication).

## Conclusion

In conclusion, our observations suggest the possibility that HCG1745306 isoform CRA-a, and histone H1.2, irrespective of their origin, could represent potential biomarkers for the detection of early ER+ BC. Further work in a larger cohort of patients is clearly needed to confirm these initial findings.

## Data availability

The data referenced by this article are under copyright with the following copyright statement: Copyright: © 2018 Tucker R and Pedro A

Data associated with the article are available under the terms of the Creative Commons Zero "No rights reserved" data waiver (CC0 1.0 Public domain dedication). 

Dataset 1: The mass spectrometry analysis results from all patient samples 10.5256/f1000research.14129.d203204 ^35^
