# Supplementary material for: Blood-derived non-extracellular vesicle proteins as potential biomarkers for the diagnosis of early ER+ breast cancer and detection of lymph node involvement
Source: F1000Res. 2018 May 10;7:283. Originally published 2018 Mar 6. [Version 3] doi: 10.12688/f1000research.14129.3 (PMC6008847; doi:10.12688/f1000research.14129.3)
Supplement: The mass spectrometry analysis results from all patient samples [file f1000research-7-16246-s0000.tgz › cde0d3c7-f158-4a00-af8f-248668b16614_dataset_1_revised.zip!/Files version 3/NTA files/1-1000 C7.pdf]

NANOSIGHT

Capture 2015-07-22 20-23-06

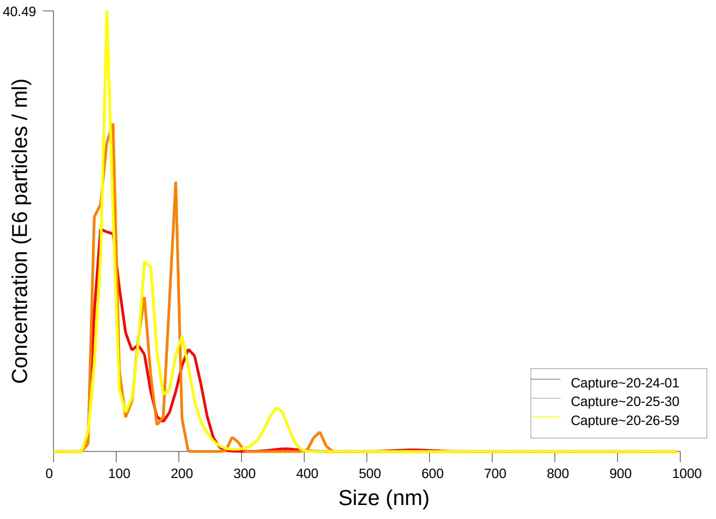

FTLA Size / Concentration graph for Experiment:
Capture 2015-07-22 20-23-06

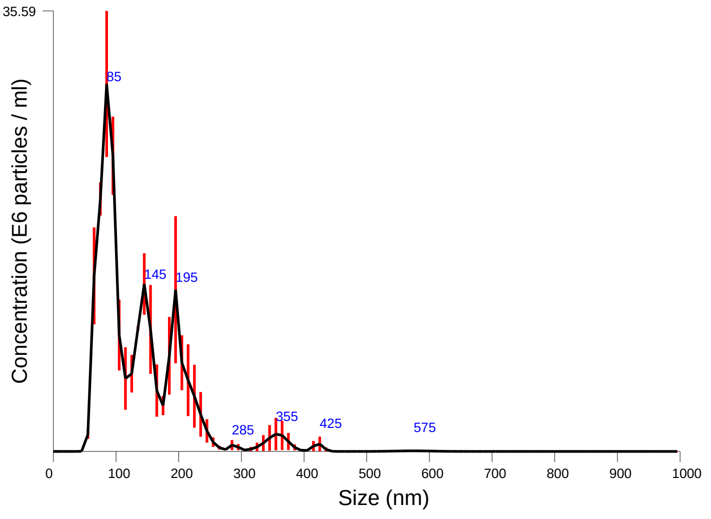

Averaged FTLA Size / Concentration
Red error bars indicate +/- 1 standard error of the mean

Included Files

Capture 2015-07-22 20-24-01
Capture 2015-07-22 20-25-30
Capture 2015-07-22 20-26-59

Details

NTA Version:	NTA 3.0 0064
Script Used:	[Modified] SOP Quick Measurement 03-11-56PM 29Apr2015.txt
Time Captured:	20:23:06 22/07/2015
Operator:	Alberto
Pre-treatment:	
Sample Name:	C7 - 1:1000
Diluent:	PBS
Remarks:	

Capture Settings

Camera Type:	SCMOS
Camera Level:	13
Slider Shutter:	800
Slider Gain:	350
FPS	25.0
Number of Frames:	1498
Temperature:	24.8 ^{o}C
Viscosity:	(Water) 0.891 - 0.892 cP
Dilution factor:	Dilution not recorded

Analysis Settings

Detect Threshold:	5
Blur Size:	Auto
Max Jump Distance:	Auto: 13.9 - 14.9 pix

Results

Stats: Merged Data

Mean:	133.0 nm
Mode:	86.2 nm
SD:	70.9 nm
D10:	62.5 nm
D50:	106.9 nm
D90:	207.5 nm

Stats: Mean +/- Standard Error

Mean:	132.5 +/- 6.5 nm
Mode:	85.3 +/- 3.2 nm
SD:	69.6 +/- 4.3 nm
D10:	62.8 +/- 2.3 nm
D50:	107.3 +/- 11.3 nm
D90:	208.9 +/- 12.3 nm
Concentration:	2.06e+008 +/- 1.04e+007 particles/ml
	10.5 +/- 0.5 particles/frame
	11.0 +/- 0.7 centres/frame
