# Supplementary material for: Blood-derived non-extracellular vesicle proteins as potential biomarkers for the diagnosis of early ER+ breast cancer and detection of lymph node involvement
Source: F1000Res. 2018 May 10;7:283. Originally published 2018 Mar 6. [Version 3] doi: 10.12688/f1000research.14129.3 (PMC6008847; doi:10.12688/f1000research.14129.3)
Supplement: The mass spectrometry analysis results from all patient samples [file f1000research-7-16246-s0000.tgz › cde0d3c7-f158-4a00-af8f-248668b16614_dataset_1_revised.zip!/Files version 3/NTA files/1-1000 CF110.pdf]

NANOSIGHT

Capture 2015-07-22 20-47-57

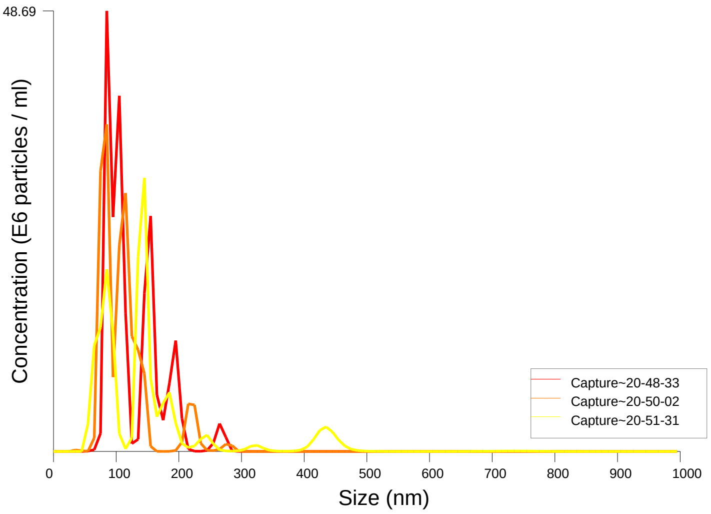

FTLA Size / Concentration graph for Experiment:
Capture 2015-07-22 20-47-57

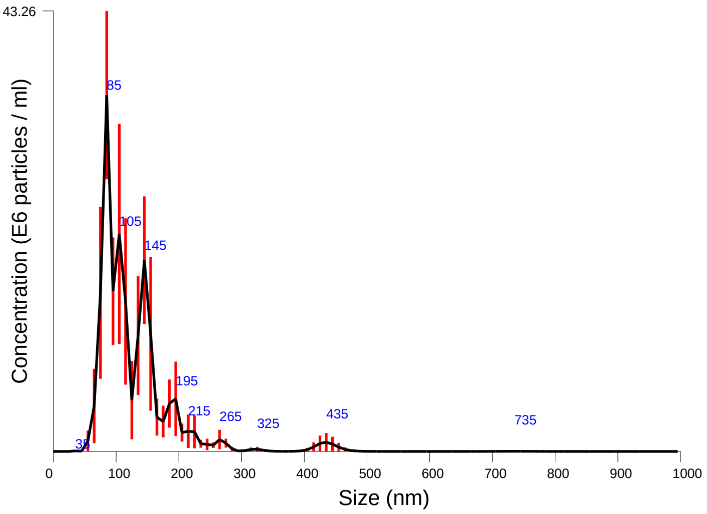

Averaged FTLA Size / Concentration
Red error bars indicate +/- 1 standard error of the mean

Included Files

Capture 2015-07-22 20-48-33
Capture 2015-07-22 20-50-02
Capture 2015-07-22 20-51-31

Details

NTA Version:	NTA 3.0 0064
Script Used:	[Modified] SOP Quick Measurement 03-11-56PM 29Apr2015.txt
Time Captured:	20:47:57 22/07/2015
Operator:	Alberto
Pre-treatment:	
Sample Name:	C110 - 1:1000
Diluent:	PBS
Remarks:	

Capture Settings

Camera Type:	SCMOS
Camera Level:	13
Slider Shutter:	800
Slider Gain:	350
FPS	25.0
Number of Frames:	1498
Temperature:	24.8 ^{o}C
Viscosity:	(Water) 0.892 - 0.892 cP
Dilution factor:	Dilution not recorded

Analysis Settings

Detect Threshold:	5
Blur Size:	Auto
Max Jump Distance:	Auto: 14.5 - 16.7 pix

Results

Stats: Merged Data

Mean:	123.7 nm
Mode:	85.0 nm
SD:	64.9 nm
D10:	68.3 nm
D50:	99.8 nm
D90:	182.6 nm

Stats: Mean +/- Standard Error

Mean:	124.4 +/- 11.2 nm
Mode:	103.7 +/- 19.6 nm
SD:	60.0 +/- 18.1 nm
D10:	66.8 +/- 3.8 nm
D50:	106.8 +/- 10.5 nm
D90:	183.4 +/- 27.8 nm
Concentration:	1.86e+008 +/- 1.57e+007 particles/ml
	9.4 +/- 0.8 particles/frame
	9.9 +/- 0.8 centres/frame
