# Supplementary material for: Blood-derived non-extracellular vesicle proteins as potential biomarkers for the diagnosis of early ER+ breast cancer and detection of lymph node involvement
Source: F1000Res. 2018 May 10;7:283. Originally published 2018 Mar 6. [Version 3] doi: 10.12688/f1000research.14129.3 (PMC6008847; doi:10.12688/f1000research.14129.3)
Supplement: The mass spectrometry analysis results from all patient samples [file f1000research-7-16246-s0000.tgz › cde0d3c7-f158-4a00-af8f-248668b16614_dataset_1_revised.zip!/Files version 3/NTA files/1-1000 CF25.pdf]

NANOSIGHT

Capture 2015-07-22 20-36-44

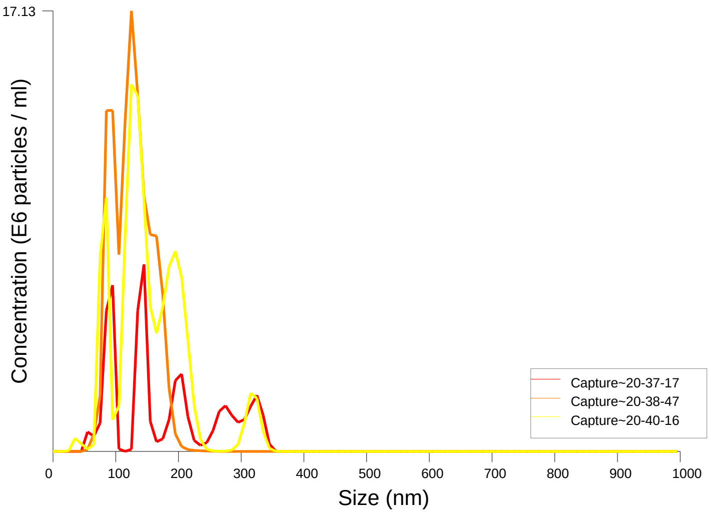

FTLA Size / Concentration graph for Experiment:
Capture 2015-07-22 20-36-44

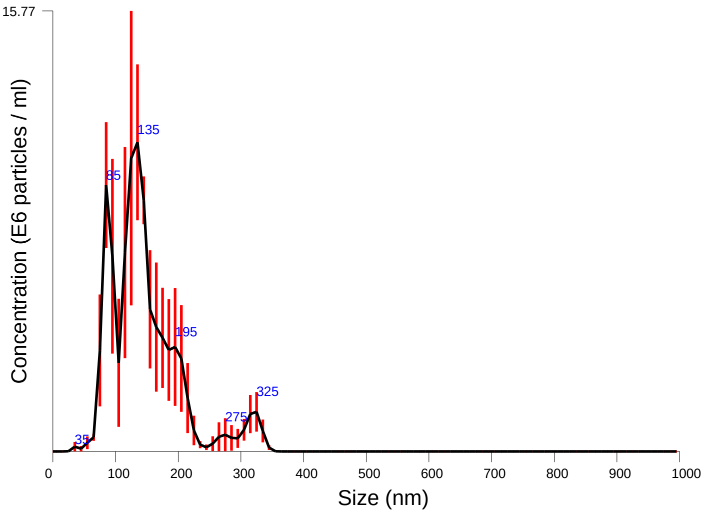

Averaged FTLA Size / Concentration
Red error bars indicate +/- 1 standard error of the mean

Included Files

Capture 2015-07-22 20-37-17
Capture 2015-07-22 20-38-47
Capture 2015-07-22 20-40-16

Details

NTA Version:	NTA 3.0 0064
Script Used:	[Modified] SOP Quick Measurement 03-11-56PM 29Apr2015.txt
Time Captured:	20:36:44 22/07/2015
Operator:	Alberto
Pre-treatment:	
Sample Name:	C25 - 1:1000
Diluent:	PBS
Remarks:	

Capture Settings

Camera Type:	SCMOS
Camera Level:	13
Slider Shutter:	800
Slider Gain:	350
FPS	25.0
Number of Frames:	1498
Temperature:	24.8 ^{o}C
Viscosity:	(Water) 0.891 - 0.892 cP
Dilution factor:	Dilution not recorded

Analysis Settings

Detect Threshold:	5
Blur Size:	Auto
Max Jump Distance:	Auto: 13.9 - 18.8 pix

Results

Stats: Merged Data

Mean:	141.8 nm
Mode:	132.1 nm
SD:	58.0 nm
D10:	75.2 nm
D50:	125.5 nm
D90:	201.0 nm

Stats: Mean +/- Standard Error

Mean:	147.7 +/- 15.3 nm
Mode:	132.4 +/- 5.0 nm
SD:	56.7 +/- 15.4 nm
D10:	75.0 +/- 1.0 nm
D50:	128.3 +/- 6.9 nm
D90:	221.1 +/- 42.8 nm
Concentration:	9.62e+007 +/- 2.19e+007 particles/ml
	4.9 +/- 1.1 particles/frame
	6.9 +/- 1.8 centres/frame
