# Supplementary material for: Blood-derived non-extracellular vesicle proteins as potential biomarkers for the diagnosis of early ER+ breast cancer and detection of lymph node involvement
Source: F1000Res. 2018 May 10;7:283. Originally published 2018 Mar 6. [Version 3] doi: 10.12688/f1000research.14129.3 (PMC6008847; doi:10.12688/f1000research.14129.3)
Supplement: The mass spectrometry analysis results from all patient samples [file f1000research-7-16246-s0000.tgz › cde0d3c7-f158-4a00-af8f-248668b16614_dataset_1_revised.zip!/Files version 3/NTA files/1-1000 CF37.pdf]

NANOSIGHT

Capture 2015-07-22 21-11-43

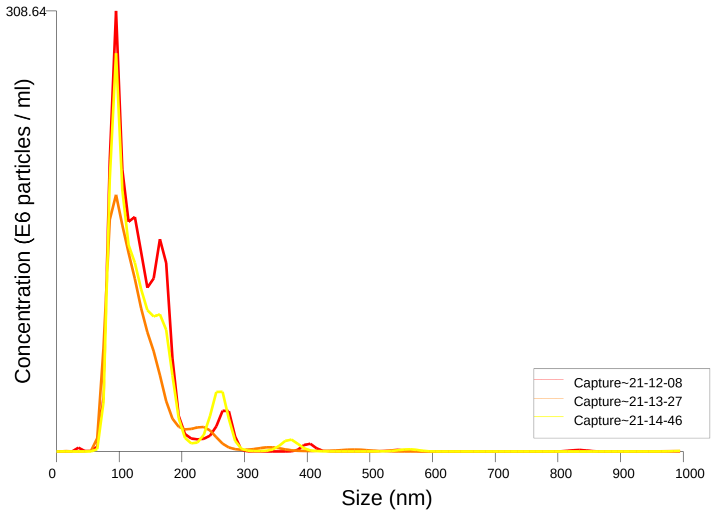

FTLA Size / Concentration graph for Experiment:
Capture 2015-07-22 21-11-43

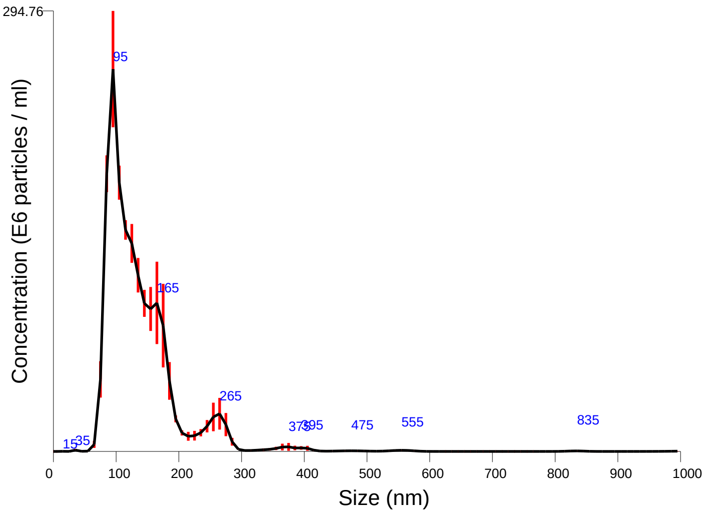

Averaged FTLA Size / Concentration
Red error bars indicate +/- 1 standard error of the mean

Included Files

Capture 2015-07-22 21-12-08
Capture 2015-07-22 21-13-27
Capture 2015-07-22 21-14-46

Details

NTA Version:	NTA 3.0 0064
Script Used:	[Modified] SOP Quick Measurement 03-11-56PM 29Apr2015.txt
Time Captured:	21:11:43 22/07/2015
Operator:	Alberto
Pre-treatment:	
Sample Name:	C37 - 1:1000
Diluent:	PBS
Remarks:	

Capture Settings

Camera Type:	SCMOS
Camera Level:	13
Slider Shutter:	800
Slider Gain:	350
FPS	25.0
Number of Frames:	1498
Temperature:	24.7 ^{o}C
Viscosity:	(Water) 0.9 cP
Dilution factor:	Dilution not recorded

Analysis Settings

Detect Threshold:	5
Blur Size:	Auto
Max Jump Distance:	Auto: 14.2 pix

Results

Stats: Merged Data

Mean:	132.7 nm
Mode:	94.8 nm
SD:	64.1 nm
D10:	76.2 nm
D50:	111.6 nm
D90:	188.2 nm

Stats: Mean +/- Standard Error

Mean:	132.3 +/- 2.9 nm
Mode:	94.7 +/- 0.1 nm
SD:	64.0 +/- 2.0 nm
D10:	76.0 +/- 1.4 nm
D50:	111.4 +/- 2.4 nm
D90:	198.2 +/- 17.0 nm
Concentration:	1.69e+009 +/- 1.83e+008 particles/ml
	86.0 +/- 9.3 particles/frame
	99.1 +/- 10.3 centres/frame
