# Supplementary material for: Blood-derived non-extracellular vesicle proteins as potential biomarkers for the diagnosis of early ER+ breast cancer and detection of lymph node involvement
Source: F1000Res. 2018 May 10;7:283. Originally published 2018 Mar 6. [Version 3] doi: 10.12688/f1000research.14129.3 (PMC6008847; doi:10.12688/f1000research.14129.3)
Supplement: The mass spectrometry analysis results from all patient samples [file f1000research-7-16246-s0000.tgz › cde0d3c7-f158-4a00-af8f-248668b16614_dataset_1_revised.zip!/Files version 3/NTA files/CF1.pdf]

NANOSIGHT

Capture 2015-07-22 19-53-27

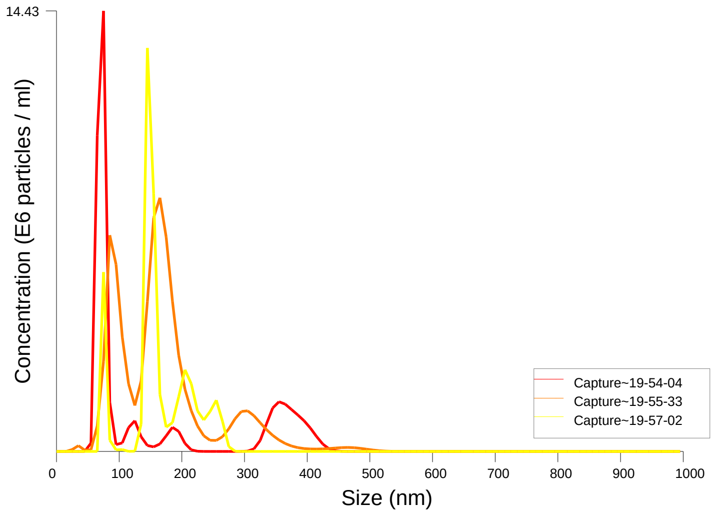

FTLA Size / Concentration graph for Experiment:
Capture 2015-07-22 19-53-27

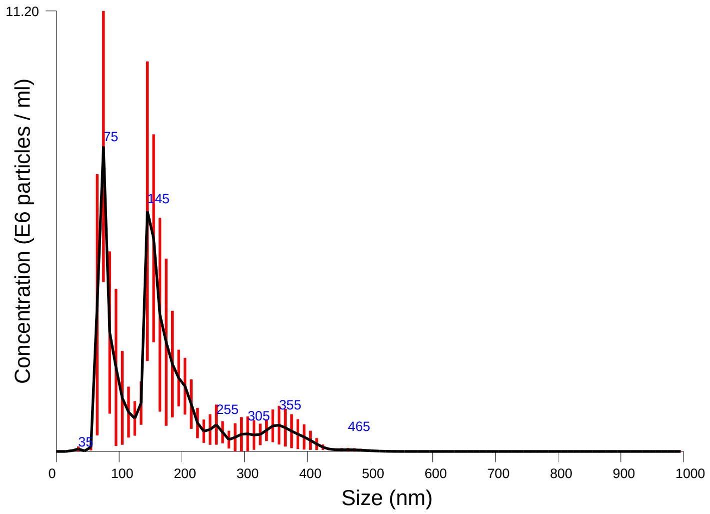

Averaged FTLA Size / Concentration
Red error bars indicate +/- 1 standard error of the mean

Included Files

Capture 2015-07-22 19-54-04
Capture 2015-07-22 19-55-33
Capture 2015-07-22 19-57-02

Details

NTA Version:	NTA 3.0 0064
Script Used:	[Modified] SOP Quick Measurement 03-11-56PM 29Apr2015.txt
Time Captured:	19:53:27 22/07/2015
Operator:	Alberto
Pre-treatment:	
Sample Name:	CF-1
Diluent:	PBS
Remarks:	

Capture Settings

Camera Type:	SCMOS
Camera Level:	13
Slider Shutter:	800
Slider Gain:	350
FPS	25.0
Number of Frames:	1498
Temperature:	24.7 ^{o}C
Viscosity:	(Water) 0.9 cP
Dilution factor:	Dilution not recorded

Analysis Settings

Detect Threshold:	5
Blur Size:	Auto
Max Jump Distance:	Auto: 14.2 - 19.8 pix

Results

Stats: Merged Data

Mean:	158.9 nm
Mode:	74.6 nm
SD:	87.7 nm
D10:	62.2 nm
D50:	141.2 nm
D90:	298.4 nm

Stats: Mean +/- Standard Error

Mean:	157.8 +/- 2.7 nm
Mode:	127.7 +/- 28.0 nm
SD:	84.9 +/- 23.1 nm
D10:	65.7 +/- 6.3 nm
D50:	120.1 +/- 26.3 nm
D90:	291.1 +/- 40.8 nm
Concentration:	5.60e+007 +/- 1.15e+007 particles/ml
	2.8 +/- 0.6 particles/frame
	3.8 +/- 1.0 centres/frame
