# Supplementary material for: Blood-derived non-extracellular vesicle proteins as potential biomarkers for the diagnosis of early ER+ breast cancer and detection of lymph node involvement
Source: F1000Res. 2018 May 10;7:283. Originally published 2018 Mar 6. [Version 3] doi: 10.12688/f1000research.14129.3 (PMC6008847; doi:10.12688/f1000research.14129.3)
Supplement: The mass spectrometry analysis results from all patient samples [file f1000research-7-16246-s0000.tgz › cde0d3c7-f158-4a00-af8f-248668b16614_dataset_1_revised.zip!/Files version 3/NTA files/CF110.pdf]

NANOSIGHT

Capture 2015-07-22 20-06-13

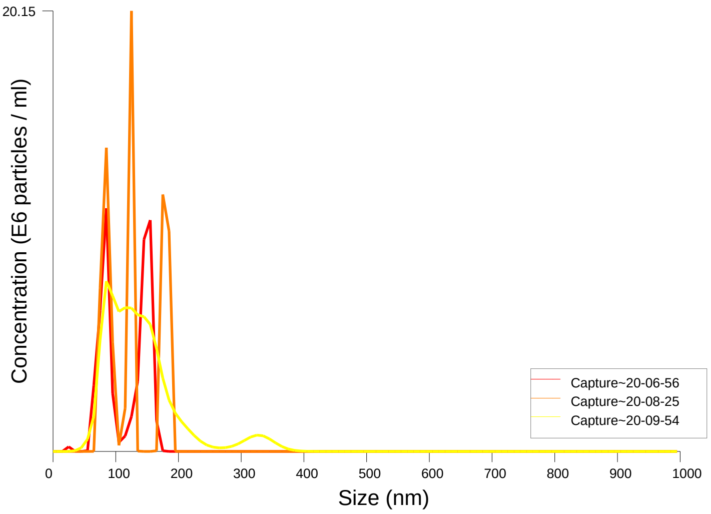

FTLA Size / Concentration graph for Experiment:
Capture 2015-07-22 20-06-13

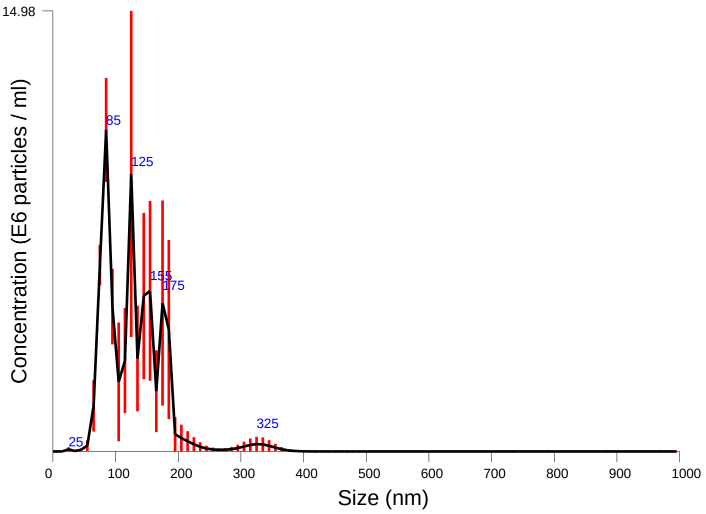

Averaged FTLA Size / Concentration
Red error bars indicate +/- 1 standard error of the mean

Included Files

Capture 2015-07-22 20-06-56
Capture 2015-07-22 20-08-25
Capture 2015-07-22 20-09-54

Details

NTA Version:	NTA 3.0 0064
Script Used:	[Modified] SOP Quick Measurement 03-11-56PM 29Apr2015.txt
Time Captured:	20:06:13 22/07/2015
Operator:	Alberto
Pre-treatment:	
Sample Name:	CF-110
Diluent:	PBS
Remarks:	

Capture Settings

Camera Type:	SCMOS
Camera Level:	14
Slider Shutter:	1000
Slider Gain:	400
FPS	25.0
Number of Frames:	1498
Temperature:	24.8 - 24.8 ^{o}C
Viscosity:	(Water) 0.9 cP
Dilution factor:	Dilution not recorded

Analysis Settings

Detect Threshold:	5
Blur Size:	Auto
Max Jump Distance:	Auto: 14.2 - 17.4 pix

Results

Stats: Merged Data	
Mean:	124.1 nm
Mode:	84.3 nm
SD:	49.4 nm
D10:	67.7 nm
D50:	114.6 nm
D90:	172.3 nm

Stats: Mean +/- Standard Error	
Mean:	122.4 +/- 7.0 nm
Mode:	98.8 +/- 13.1 nm
SD:	45.2 +/- 8.1 nm
D10:	67.7 +/- 2.5 nm
D50:	115.8 +/- 1.4 nm
D90:	172.0 +/- 14.4 nm
Concentration:	6.77e+007 +/- 8.83e+006 particles/ml
	3.4 +/- 0.4 particles/frame
	4.6 +/- 1.0 centres/frame
