# Supplementary material for: Blood-derived non-extracellular vesicle proteins as potential biomarkers for the diagnosis of early ER+ breast cancer and detection of lymph node involvement
Source: F1000Res. 2018 May 10;7:283. Originally published 2018 Mar 6. [Version 3] doi: 10.12688/f1000research.14129.3 (PMC6008847; doi:10.12688/f1000research.14129.3)
Supplement: The mass spectrometry analysis results from all patient samples [file f1000research-7-16246-s0000.tgz › cde0d3c7-f158-4a00-af8f-248668b16614_dataset_1_revised.zip!/Files version 3/NTA files/CF27.pdf]

NANOSIGHT

Capture 2015-05-29 16-45-06

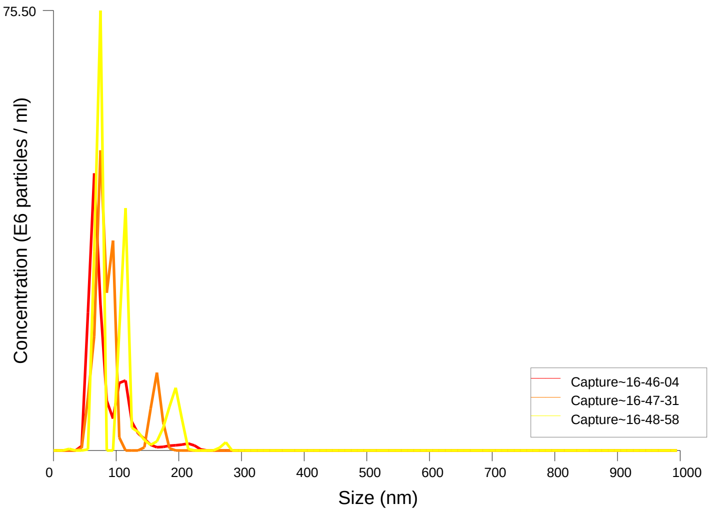

FTLA Size / Concentration graph for Experiment:
Capture 2015-05-29 16-45-06

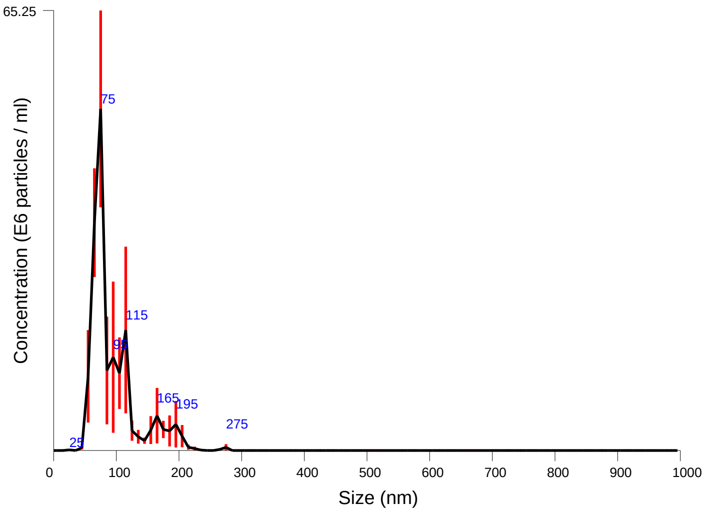

Averaged FTLA Size / Concentration
Red error bars indicate +/- 1 standard error of the mean

Included Files

Capture 2015-05-29 16-46-04
Capture 2015-05-29 16-47-31
Capture 2015-05-29 16-48-58

Details

NTA Version:	NTA 3.0 0064
Script Used:	[Modified] 3 x 60s capture sequence, no autosampler.txt
Time Captured:	16:45:06 29/05/2015
Operator:	Alberto
Pre-treatment:	
Sample Name:	Ana - Cf 27
Diluent:	
Remarks:	

Capture Settings

Camera Type:	SCMOS
Camera Level:	13
Slider Shutter:	800
Slider Gain:	350
FPS	25.0
Number of Frames:	1498
Temperature:	21.0 - 21.0 °C
Viscosity:	(Water) 0.976 - 0.976 cP
Dilution factor:	Dilution not recorded

Analysis Settings

Detect Threshold:	5
Blur Size:	Auto
Max Jump Distance:	Auto: 15.6 - 16.7 pix

Results

Stats: Merged Data

Mean:	90.0 nm
Mode:	73.0 nm
SD:	38.5 nm
D10:	51.9 nm
D50:	68.7 nm
D90:	151.5 nm

Stats: Mean +/- Standard Error

Mean:	88.8 +/- 5.7 nm
Mode:	71.4 +/- 3.2 nm
SD:	36.6 +/- 3.6 nm
D10:	52.0 +/- 3.0 nm
D50:	67.7 +/- 3.2 nm
D90:	146.9 +/- 18.1 nm
Concentration:	1.79e+008 +/- 1.82e+007 particles/ml
	9.1 +/- 0.9 particles/frame
	9.7 +/- 1.1 centres/frame
