# Supplementary material for: Blood-derived non-extracellular vesicle proteins as potential biomarkers for the diagnosis of early ER+ breast cancer and detection of lymph node involvement
Source: F1000Res. 2018 May 10;7:283. Originally published 2018 Mar 6. [Version 3] doi: 10.12688/f1000research.14129.3 (PMC6008847; doi:10.12688/f1000research.14129.3)
Supplement: The mass spectrometry analysis results from all patient samples [file f1000research-7-16246-s0000.tgz › cde0d3c7-f158-4a00-af8f-248668b16614_dataset_1_revised.zip!/Files version 3/NTA files/CF33.pdf]

NANOSIGHT

(null) 2015-05-29 17-07-52

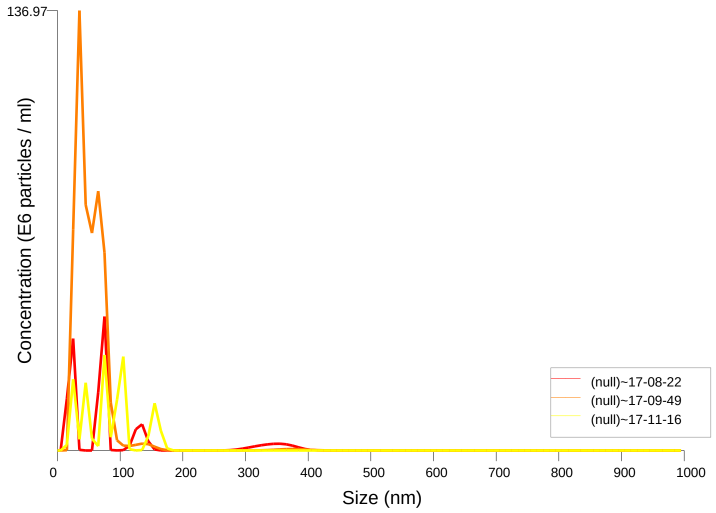

FTLA Size / Concentration graph for Experiment:
(null) 2015-05-29 17-07-52

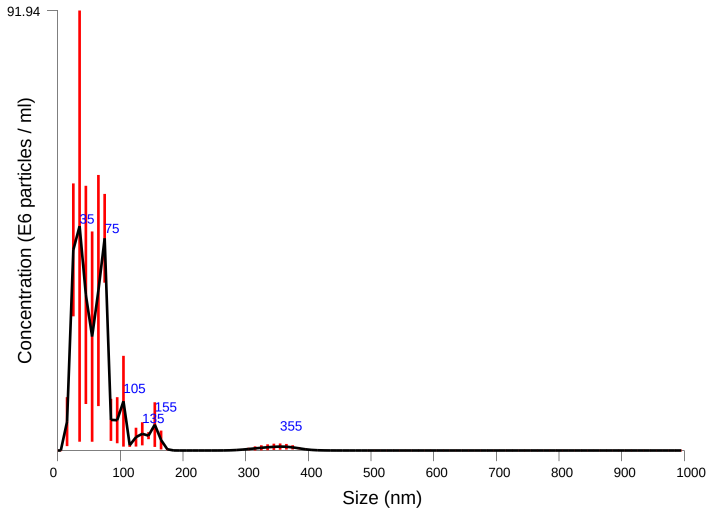

Averaged FTLA Size / Concentration
Red error bars indicate +/- 1 standard error of the mean

Included Files

(null) 2015-05-29 17-08-22
(null) 2015-05-29 17-09-49
(null) 2015-05-29 17-11-16

Details

NTA Version:	NTA 3.0 0064
Script Used:	[Modified] 3 x 60s capture sequence, no autosampler.txt
Time Captured:	17:07:52 29/05/2015
Operator:	Alberto
Pre-treatment:	
Sample Name:	Ana - Cf 33
Diluent:	
Remarks:	

Capture Settings

Camera Type:	SCMOS
Camera Level:	13
Slider Shutter:	800
Slider Gain:	350
FPS	25.0
Number of Frames:	1498
Temperature:	21.4 - 21.4 ^{o}C
Viscosity:	(Water) 0.966 - 0.967 cP
Dilution factor:	Dilution not recorded

Analysis Settings

Detect Threshold:	1
Blur Size:	Auto
Max Jump Distance:	Auto: 14.2 - 22.1 pix

Results

Stats: Merged Data

Mean:	60.5 nm
Mode:	32.6 nm
SD:	53.8 nm
D10:	15.2 nm
D50:	44.6 nm
D90:	97.1 nm

Stats: Mean +/- Standard Error

Mean:	71.0 +/- 12.0 nm
Mode:	61.4 +/- 13.1 nm
SD:	56.6 +/- 19.8 nm
D10:	14.7 +/- 2.4 nm
D50:	55.7 +/- 9.4 nm
D90:	168.0 +/- 66.6 nm
Concentration:	2.77e+008 +/- 1.24e+008 particles/ml
	14.0 +/- 6.3 particles/frame
	349.1 +/- 156.2 centres/frame
