# Supplementary material for: Blood-derived non-extracellular vesicle proteins as potential biomarkers for the diagnosis of early ER+ breast cancer and detection of lymph node involvement
Source: F1000Res. 2018 May 10;7:283. Originally published 2018 Mar 6. [Version 3] doi: 10.12688/f1000research.14129.3 (PMC6008847; doi:10.12688/f1000research.14129.3)
Supplement: The mass spectrometry analysis results from all patient samples [file f1000research-7-16246-s0000.tgz › cde0d3c7-f158-4a00-af8f-248668b16614_dataset_1_revised.zip!/Files version 3/NTA files/CF37.pdf]

NANOSIGHT

Capture 2015-07-22 19-40-56

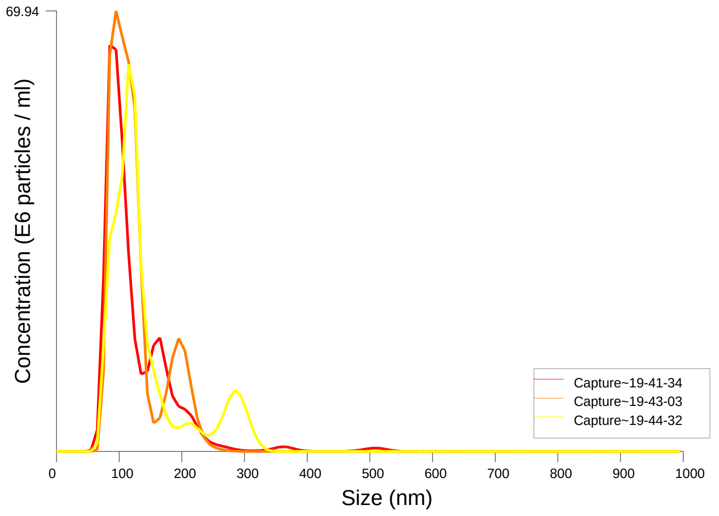

FTLA Size / Concentration graph for Experiment:
Capture 2015-07-22 19-40-56

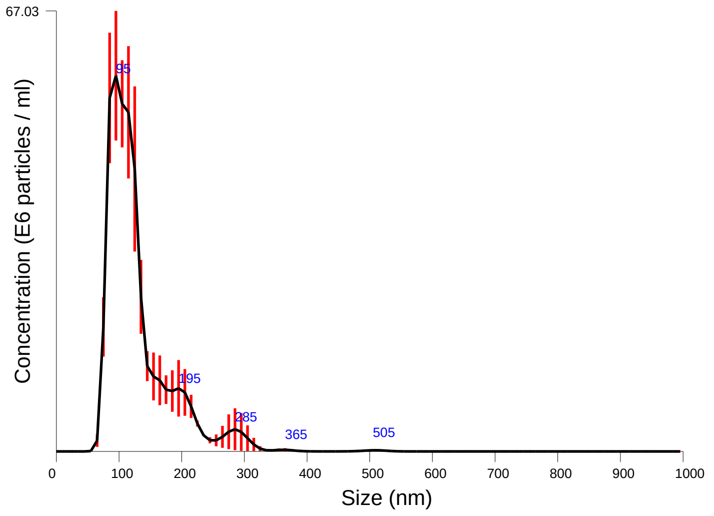

Averaged FTLA Size / Concentration
Red error bars indicate +/- 1 standard error of the mean

Included Files

Capture 2015-07-22 19-41-34
Capture 2015-07-22 19-43-03
Capture 2015-07-22 19-44-32

Details

NTA Version:	NTA 3.0 0064
Script Used:	[Modified] SOP Quick Measurement 03-11-56PM 29Apr2015.txt
Time Captured:	19:40:56 22/07/2015
Operator:	Alberto
Pre-treatment:	
Sample Name:	CF-37
Diluent:	PBS
Remarks:	

Capture Settings

Camera Type:	SCMOS
Camera Level:	13
Slider Shutter:	800
Slider Gain:	350
FPS	25.0
Number of Frames:	1498
Temperature:	24.7 ^{o}C
Viscosity:	(Water) 0.9 cP
Dilution factor:	Dilution not recorded

Analysis Settings

Detect Threshold:	5
Blur Size:	Auto
Max Jump Distance:	Auto: 12.8 - 14.4 pix

Results

Stats: Merged Data

Mean:	124.8 nm
Mode:	94.4 nm
SD:	53.6 nm
D10:	73.8 nm
D50:	103.9 nm
D90:	192.3 nm

Stats: Mean +/- Standard Error

Mean:	125.0 +/- 5.6 nm
Mode:	101.3 +/- 8.5 nm
SD:	52.8 +/- 6.8 nm
D10:	74.2 +/- 1.6 nm
D50:	103.0 +/- 4.3 nm
D90:	206.2 +/- 24.7 nm
Concentration:	4.09e+008 +/- 2.31e+007 particles/ml
	20.8 +/- 1.2 particles/frame
	21.5 +/- 1.1 centres/frame
