# Supplementary material for: Blood-derived non-extracellular vesicle proteins as potential biomarkers for the diagnosis of early ER+ breast cancer and detection of lymph node involvement
Source: F1000Res. 2018 May 10;7:283. Originally published 2018 Mar 6. [Version 3] doi: 10.12688/f1000research.14129.3 (PMC6008847; doi:10.12688/f1000research.14129.3)
Supplement: The mass spectrometry analysis results from all patient samples [file f1000research-7-16246-s0000.tgz › cde0d3c7-f158-4a00-af8f-248668b16614_dataset_1_revised.zip!/Files version 3/NTA files/CF5.pdf]

NANOSIGHT

Capture 2015-07-22 18-55-26

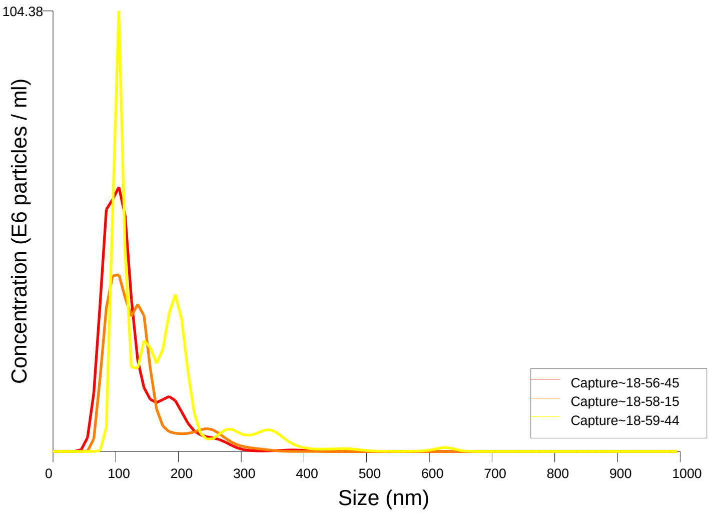

FTLA Size / Concentration graph for Experiment:
Capture 2015-07-22 18-55-26

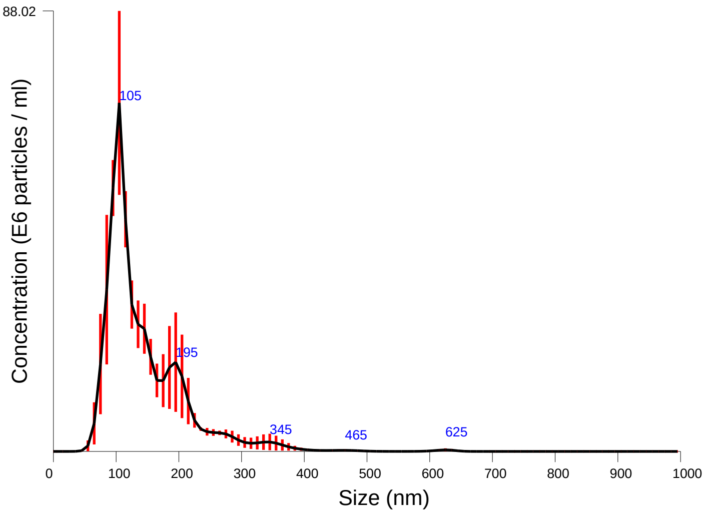

Averaged FTLA Size / Concentration
Red error bars indicate +/- 1 standard error of the mean

Included Files

Capture 2015-07-22 18-56-45
Capture 2015-07-22 18-58-15
Capture 2015-07-22 18-59-44

Details

NTA Version:	NTA 3.0 0064
Script Used:	[Modified] SOP Quick Measurement 03-11-56PM 29Apr2015.txt
Time Captured:	18:55:26 22/07/2015
Operator:	Alberto
Pre-treatment:	
Sample Name:	CF-5
Diluent:	PBS
Remarks:	

Capture Settings

Camera Type:	SCMOS
Camera Level:	13
Slider Shutter:	800
Slider Gain:	350
FPS	25.0
Number of Frames:	1498
Temperature:	24.7 ^{o}C
Viscosity:	(Water) 0.894 - 0.895 cP
Dilution factor:	Dilution not recorded

Analysis Settings

Detect Threshold:	5
Blur Size:	Auto
Max Jump Distance:	Auto: 12.3 - 14.5 pix

Results

Stats: Merged Data

Mean:	140.0 nm
Mode:	104.2 nm
SD:	68.4 nm
D10:	76.7 nm
D50:	111.5 nm
D90:	213.4 nm

Stats: Mean +/- Standard Error

Mean:	137.7 +/- 13.5 nm
Mode:	102.7 +/- 1.2 nm
SD:	61.4 +/- 10.6 nm
D10:	77.2 +/- 6.1 nm
D50:	117.0 +/- 11.5 nm
D90:	220.9 +/- 25.0 nm
Concentration:	4.60e+008 +/- 5.53e+007 particles/ml
	23.3 +/- 2.8 particles/frame
	25.3 +/- 2.5 centres/frame
