# Supplementary material for: Blood-derived non-extracellular vesicle proteins as potential biomarkers for the diagnosis of early ER+ breast cancer and detection of lymph node involvement
Source: F1000Res. 2018 May 10;7:283. Originally published 2018 Mar 6. [Version 3] doi: 10.12688/f1000research.14129.3 (PMC6008847; doi:10.12688/f1000research.14129.3)
Supplement: Peer review history

# Peer review: Referee response for version 3

**DOI:** 10.5256/f1000research.16246.r33915

**Published:** 2018-06-18

**Authors:** Matthew J. Shurtleff

**Affiliations:** 1 University of California, San Francisco (UCSF), San Francisco, CA, USA

The results and conclusions are improved from previous revisions. However, as written, the paper is non-sensical. The title now states that the biomarkers are non-EV proteins, and the results and conclusions are appropriately skeptical about the EV origin of these proteins. However, the introduction only discusses the promise of EVs as biomarkers (and seems substantially unchanged from previous versions). I think any reader would find the introduction to be quite disconnected from the revised title, results and conclusions. For clarity, and to appropriately contextualize the study, the introduction should be revised to focus on the utility of circulating biomarkers, irregardless of their association with EVs.

I have read this submission. I believe that I have an appropriate level of expertise to confirm that it is of an acceptable scientific standard.

# Peer review: Referee response for version 2

**DOI:** 10.5256/f1000research.16084.r33412

**Published:** 2018-05-01

**Authors:** Matthew J. Shurtleff

**Affiliations:** 1 University of California, San Francisco (UCSF), San Francisco, CA, USA

The authors have not made a true attempt to satisfy my reservations. They have only parroted my reservations in their response and discussion, and removed the word "vesicle" from their title. They have not attempted to revise the content of their article at all. Indeed, the first two words of the text remain "extracellular vesicles". I do not consider this work satisfactory for indexing.

I have read this submission. I believe that I have an appropriate level of expertise to state that I do not consider it to be of an acceptable scientific standard, for reasons outlined above.

# Peer review: Referee response for version 1

**DOI:** 10.5256/f1000research.15368.r32581

**Published:** 2018-04-16

**Authors:** Matthew J. Shurtleff

**Affiliations:** 1 University of California, San Francisco (UCSF), San Francisco, CA, USA

Tucker and Pedro present results from proteomics studies of material precipitated from the plasma of breast cancer patients and healthy controls, and from the media of explant cultures and cell lines. They observe CRA-a (HBA2, alpha hemoglobin) as being present in precipitated material from the plasma of patients with early breast cancer and Histone H1.2 as being present in samples with lymph node involvement (plasma), and the media of explant cultures of bone metastases and metastatic cell lines.

 While the results are clearly presented, the interpretation that these are EV-based biomarkers is not supported. The EV isolation method used is insufficient to purify EVs from other contaminants. Furthermore, it is not intuitive that EVs would be likely to contain a histone (normally confined to the nucleus) or hemoglobin. The authors' discussion of this major concern is insufficient ("However, samples submitted for mass spectrometry analysis showed none of the recognised contaminants and it was therefore concluded that the main EV type present in these samples is the MV.").

 The EV literature is quite confusing due, in part, to the over-interpretation of observations from samples prepared using inadequate isolation methods. Therefore, in it's current version, I do not recommend indexing of this report. Ideally, the authors should repeat the work with approaches that better enrich for EVs over non-EV protein contaminants (e.g. density-gradient based ultracentrifugation or immuno-affinity purification) and protease protection assays in the presence/absence of detergent should be used evaluate if the proteins are indeed associated with EVs and if they are on the surface or interior of vesicles. If further experiments are not possible, I recommend revising the paper to remove the unsupported conclusion that the proteins identified are EV-associated. (For example the title could be: Blood-derived extracellular proteins as potential biomarkers for the diagnosis of early ER+ breast cancer and detection of lymph node involvement).

 For reference, I direct the authors to an updated publication prepared by members of the EV community on the minimal information for studies of EVs in the Journal of Extracellular Vesicles ^1^.

I have read this submission. I believe that I have an appropriate level of expertise to confirm that it is of an acceptable scientific standard, however I have significant reservations, as outlined above.

## References

[1] WitwerKSoekmadjiCHillAWaubenMBuzásEDi VizioDFalcon-PerezJGardinerCHochbergFKurochkinILötvallJMathivananSNieuwlandRSahooSTaharaHTorrecilhasAWeaverAYinHZhengLGhoYQuesenberryPThéryC: Updating the MISEV minimal requirements for extracellular vesicle studies: building bridges to reproducibility. *Journal of Extracellular Vesicles*.2017;6(1) : 10.1080/20013078.2017.1396823 10.1080/20013078.2017.1396823 PMC569893729184626

# Peer review: Referee response for version 1

**DOI:** 10.5256/f1000research.15368.r32046

**Published:** 2018-03-26

**Authors:** Shweta Aras

**Affiliations:** 1 Lewis Katz School of Medicine, Temple University, Philadelphia, PA, USA

In this research note by Tucker et al., authors have identified 2 novel biomarkers potentially involved in early breast cancer development as well as as lymph node metastasis using extracellular vesicles from patient samples along with adequate controls of metastatic and non-metastatic cell lines. Several reports in the literature have already suggested the importance of EVs in cancer initiation and progression in various types of cancers. Although the novelty of this publication is limited, identification of 2 new molecules differentially expressed in primary versus metastatic breast cancer patients opens up the possibility of them being used in a simple prognostic blood test for detecting early BC development as opposed to mammography and other invasive techniques. A probable limitation of the study would be lack of identification of molecular mechanisms through which this upregulation of CRA-a and histone H1.2 phosphorylation plays a role in metastasis, specifically in which step of metastatic cascade e.g. intravasation, EMT, extravasation etc? But overall, the authors have done a great job and the article is a good addition to the field.

I have read this submission. I believe that I have an appropriate level of expertise to confirm that it is of an acceptable scientific standard.
